# Application of chronic liver failure-sequential organ failure assessment score for the predication of mortality after esophageal variceal hemorrhage post endoscopic ligation

**DOI:** 10.1371/journal.pone.0182529

**Published:** 2017-08-02

**Authors:** Ming-Wun Wong, Ming-Jen Chen, Huan-Lin Chen, Yu-Chi Kuo, I-Tsung Lin, Chia-Hsien Wu, Yuan-Kai Lee, Chun-Han Cheng, Ming-Jong Bair

**Affiliations:** 1 Department of Medicine, Buddhist Tzu Chi General Hospital and Tzu Chi University, Hualien, Taiwan; 2 Department of Internal Medicine, Division of Gastroenterology, Taitung MacKay Memorial Hospital, Taitung, Taiwan; 3 Department of Internal Medicine, Division of Gastroenterology, MacKay Memorial Hospital, Taipei, Taiwan; 4 MacKay Medicine Nursing and Management College, Taipei, Taiwan; 5 MacKay Medical College, New Taipei, Taiwan; 6 Department of Nursing, Meiho University, Pingtung, Taiwan; Taipei Veterans General Hospital, TAIWAN

## Abstract

**Background:**

Esophageal variceal hemorrhage (EVH) is one of the high mortality complications in cirrhotic patients. Endoscopic variceal ligation (EVL) is currently the standard therapy for EVH. However, some patients have expired during hospitalization or survived shortly after management.

**Aim:**

To evaluate hospital and 6-week mortality by receiver operating characteristic (ROC) curve of chronic liver failure-sequential organ failure assessment (CLIF-SOFA) score compared to a model for end-stage liver disease (MELD) score and Child–Turcotte–Pugh (CTP) class.

**Methods:**

We retrospectively collected 714 cirrhotic patients with EVH post EVL between July 2010 and June 2016 at Taitung MacKay Memorial Hospital, Taiwan. CLIF-SOFA score, MELD score, and CTP class were calculated for all patients admitted.

**Results:**

Among the 714 patients, the overall hospital and 6-week mortality rates were 6.9% (49/715) and 13.1% (94/715) respectively. For predicting hospital death, area under receiver operating characteristic curve (AUROC) values of CLIF-SOFA score, MELD score, and CTP class were 0.964, 0.876, and 0.846. For predicting 6-week death, AUROC values of CLIF-SOFA score, MELD score, and CTP class were 0.943, 0.817, and 0.834. CLIF-SOFA score had higher AUROC value with statistical significance under pairwise comparison than did MELD score and CTP class in prediction of not only hospital but also 6-week mortality. The history of hepatocellular carcinoma was the risk factor for 6-week mortality. For patients with hepatocellular carcinoma the cut-point of CLIF-SOFA score was 5.5 for 6-week mortality and 6.5 for hospital mortality on admission. For patients without hepatocellular carcinoma, the cut-point of CLIF-SOFA score was 6.5 for both 6-week and hospital mortality.

**Conclusion:**

CLIF-SOFA score predicted post-EVL prognosis well. For patients without hepatocellular carcinoma, CLIF-SOFA score ≥6 suggests higher 6-week mortality and CLIF-SOFA score ≥7 suggests higher hospital mortality. For patients with hepatocellular carcinoma, CLIF-SOFA score ≥7 suggests higher 6-week and hospital mortality.

## Introduction

Acute esophageal variceal hemorrhage (EVH) is one of the lethal complications in patients with liver cirrhosis. Endoscopic variceal ligation (EVL) is currently the standard therapy for acute EVH[1]. Although patient survival has improved under advanced endoscopy, antibiotic prophylaxis, and vasoactive medication, the mortality rate is still up to 20% for each episode of acute variceal hemorrhage[2–5]. Some patients have died later even though the initial EVL was successful. Therefore, according to the Baveno VI consensus, 6-week mortality was suggested for primary endpoint of acute variceal hemorrhage. In addition, Child–Turcotte–Pugh (CTP) class, end-stage liver disease (MELD) score, and primary hemostasis were useful for predicting 6-week mortality[6].

The sequential organ failure assessment (SOFA) score was originally developed for predicting the outcome of patients in intensive care units(ICU)[7]. The chronic liver failure-sequential organ failure assessment (CLIF-SOFA) score (S1 Table) was adjusted from SOFA score by assessing the six organ systems including the liver (bilirubin level), cerebral function (hepatic encephalopathy grade), coagulation (international normalized ratio(INR)), circulation (mean arterial pressure), and lung (PaO2/FiO2 or SpO2/FiO2)[8].

We aimed to evaluate predictive power of CLIF-SOFA score for 6-week and hospital mortality of patients with EVH post EVL. We also compared the result with CTP class and MELD score.

## Materials and methods

### Subjects

This study was conducted in accordance with the Declaration of Helsinki and was approved by the Institutional Review Board of Mackay Memorial Hospital. The patient records and information were anonymized and de-identified prior to analysis.

Seven hundred fourteen consecutive patients who underwent EVL for EVH at a single tertiary center between July 2010 and June 2016 were included in this study. All patients took terlipressin on the diagnosis of EVH[6]. The following patients were excluded: those with no evidence of liver cirrhosis, initial EVL failure, EVH managed by other techniques such as tissue adhesive therapy, balloon tamponade, or transjugular intrahepatic portacaval shunt.

Demographic data, disease history such as the cause of liver cirrhosis and hepatocellular carcinoma were obtained from the hospital medical registry. The complete blood counts, platelet count, bilirubin, INR, albumin, and creatinine levels were evaluated on the same day that the patients underwent endoscopic variceal ligation. CTP class was evaluated by the Pugh modification[9]. MELD score was calculated by United Network of Organ Sharing (UNOS) adjustments[10]. CLIF-SOFA score was established by EASL-CLIF consortium[8].

### Definitions and outcomes

The EVH was defined as active bleeding on the esophageal varices or adherent clots, or white nipple signs on the esophageal varices without other sources of bleeding. The diagnosis was confirmed by endoscopy in all included patients and EVL was performed at the same time.

From the date of successful EVL, the primary endpoint of outcome was 6-week survival according to Baveno VI recommendations[6]. The secondary outcome was hospital survival. Follow-up for all patients was continued until December 31, 2016.

### Statistical analysis

Student’s t test was used to compare differences between groups for continuous variables, and the chi-square test was employed for categorical data. Risk factors for 6-week mortality were tested by univariate Cox proportional hazards model first, and statistically significant ones were analyzed by multiple logistic forward Cox regression further. We used receiver operating characteristic (ROC) curve analysis to evaluate scoring systems[11]. The cut-off points was determined by best Youden index (sensitivity + specificity—1)[12]. Kaplan–Meier survival curves were constructed and compared using the log-rank test. All the statistical tests were two-tailed with value of P < 0.05 considered significant. All statistical analyses were performed using SPSS software, version 19 (IBM SPSS Statistics, State of New York)

## Results

### Patient characteristics

A total of 714 consecutive patients were included in our study for analysis from July 2010 to June 2016. The overall hospital and 6-week mortality were 6.9% (49/715) and 13.1% (94/715), respectively. The mean age of the patients was 54.7 years and 560 patients were men (78.3%) and 155 were women (21.7%). There were 146 (21%) patients who had a history of hepatocellular carcinoma. According to 6-week mortality, demographical and clinical characteristics of survivors and nonsurvivors were summarized in Table 1. Alcoholic hepatitis accounted for 35% of cases, while a further 30% were hepatitis C virus infection cases as major causes of liver cirrhosis in our study. Except for history of hepatocellular carcinoma, there were no significant differences in age, gender, and cause of liver cirrhosis between survivors and nonsurvivors.

**Table 1:** Demographical and clinical characteristics according to 6-week mortality.

Characteristics	All patients (n = 714)	Survivors (n = 621)	Nonsurvivors (n = 93)	P-value
Age (years)	54.7 ± 12.7	54.5 ± 12.5	55.8 ± 14.2	0.380
Gender (M/F)	560/154	487/134	73/20	0.987
History of HCC (%)	146 (21)	115 (19)	31 (33)	0.001
Causes of cirrhosis				
Alcoholic	256 (35)	230 (37)	26 (28)	0.157
Hepatitis B	92 (13)	79 (13)	13 (14)	0.406
Hepatitis C	214 (30)	175 (28)	39 (42)	0.663
Alcoholic + Hepatitis B	48 (7)	45 (7)	3 (3)	0.090
Alcoholic + Hepatitis C	36 (5)	34 (6)	2 (2)	0.097
Hepatitis B + Hepatitis C	15 (2)	14 (2)	1 (1)	0.242
Alcoholic + Hepatitis B + Hepatitis C	13 (2)	10 (2)	3 (3)	0.067
Other causes*	40 (6)	34 (6)	6 (7)	0.504
MAP (mmHg)	89.8 ± 20.6	91.1 ± 20.8	81.1 ± 16.5	<0.001
Hemoglobin (g/dL)	9.4 ± 2.4	9.5 ± 2.4	8.6 ± 2.1	0.001
Leucocytes (K/dL)	8.0 ± 6.0	7.5 ± 5.8	10.8 ± 6.2	<0.001
Bilirubin (umol/L)	4.5 ± 6.0	3.7 ± 4.7	10.0 ± 9.8	<0.001
Prothrombin time INR	1.4 ± 0.7	1.4 ± 0.8	1.6 ± 0.5	0.007
Albumin (g/dL)	2.9 ± 0.7	3.0 ± 0.7	2.6 ± 0.5	<0.001
Creatinine (mg/dL)	1.4 ±1.5	1.2 ± 1.0	2.5 ± 2.9	<0.001
Platelets (K/L)	106.4 ± 68.8	103.3 ± 65.8	126.6 ± 83.7	0.012
Sodium (mmol/L)	138.7 ± 45.3	139.0 ± 48.6	136.7 ± 7.0	0.652
Hepatic encephalopathy (grade)	0.5 ± 0.9	0.4 ± 0.8	1.6 ± 1.3	<0.001
SpO2/FiO2	454.4 ± 65.7	461.1 ± 56.9	409.7 ± 96.4	<0.001
CPT points	9.3 ± 1.7	4.6 ± 1.7	9.2 ± 2.5	<0.001
MELD score	14.3 ± 8.2	12.9 ± 7.0	23.4 ± 9.7	<0.001
CLIF-SOFA score	5.1 ±2.4	4.6 ± 1.7	9.2 ± 2.5	<0.001

M, male; F, female; HCC, hepatocellular carcinoma; MAP, mean arterial pressure; INR, international normalized ratio; SpO2, pulse oximetric saturation; FiO2, fractional inspired oxygen; CTP, Child–Turcotte–Pugh; MELD, model for end-stage liver disease; CLIF-SOFA, chronic liver failure-sequential organ failure assessment.

* “Other causes” includes primary biliary cirrhosis, autoimmune hepatitis, and other unknown causes.

The proportion of CTP class C was 45.1% (323/715), mean MELD score was 14.3, and mean CLIP-SOFA score was 5.2 in this study.

### Risk factors for 6-week and hospital mortality

S2 Table indicates 12 prognostic parameters for 6-week mortality by univariate Cox proportional hazards analysis. We then further performed Cox regression multivariate analysis.

With scoring systems excluded, history of hepatocellular carcinoma, mean arterial pressure (MAP), grade of hepatic encephalopathy, SpO2/FiO2, and serum level of bilirubin, albumin, and creatinine were independent risk factors for 6-week mortality.

With scoring systems included, only CLIF-SOFA score and hepatocellular carcinoma were independent risk factors for 6-week mortality.

S3 Table indicates 11 prognostic parameters for hospital mortality by univariate Cox proportional hazards analysis. Then we performed Cox regression multivariate analysis.

With scoring systems excluded, the result was almost the same as for 6-week mortality except for hepatocellular carcinoma. The history of hepatocellular carcinoma was not the risk factor for hospital mortality after multivariate analysis.

With scoring systems included, only CLIF-SOFA score and prothrombin time international normalized ratio (INR) were independent risk factors for hospital mortality.

### Validity of the scoring systems

Table 2 summarizes the performance of different scoring systems; all three had good predictive value according to ROC. The areas under the receiver operator curve (AUROC) for predicting 6-week mortality of EVH post EVL among CLIF-SOFA score, MELD score, and CTP points were 0.943, 0.817, 0.834 respectively; the AUROC for predicting hospital mortality of EVH post EVL among CLIF-SOFA score, MELD score, and CTP points were 0.964, 0.876, 0.846 respectively. CLIF-SOFA score was the best predicative model for both hospital and 6-week mortality due to the largest AUROC with statistical significance under pairwise comparison of AUROC.

**Table 2:** Pairwise comparison of area under the receiver operator characteristics (AUROC).

Variable	AUROC	95% CI	P-value	AUROC Difference	S.E.	P
**Prediction of 6-week mortality**						
CLIF-SOFA	0.943	0.917–0.968	<0.001			
MELD	0.817	0.769–0.866	<0.001	0.126	0.0324	<0.001
CTP points	0.834	0.793–0.875	<0.001	0.109	0.0315	<0.001
**Prediction of hospital mortality**						
CLIF-SOFA	0.964	0.949–0.979	<0.001			
MELD	0.876	0.825–0.927	<0.001	0.088	0.0379	0.02
CTP points	0.846	0.799–0.894	<0.001	0.118	0.0404	0.003

CLIF-SOFA, chronic liver failure-sequential organ failure assessment; MELD, model for end-stage liver disease; CTP, Child–Turcotte–Pugh

Table 3 revealed the cut-off point in manner of Youden index for CLIF-SOFA score, MELD score, and CTP points. The sensitivity, specificity, positive predictive values, negative predictive values, and accuracy were also shown at the best cut-off point. For CLIF-SOFA score, we identified the cut-off point of 6.5 for not only hospital but also 6-week mortality.

**Table 3:** Mortality prediction of EVH post EVL.

**6-week mortality**							
Predictive factors	Cut-off point	Youden index	Sensitivity (%)	Specificity (%)	PPV	NPV	Accuracy
CLIF-SOFA	6.5	0.758	0.871	0.887	0.536	0.979	0.885
MELD score	18.5	0.513	0.688	0.824	0.370	0.946	0.806
CTP points	10.5	0.499	0.699	0.800	0.344	0.947	0.787
** Hospital mortality**							
CLIF-SOFA	6.5	0.823	0.979	0.844	0.311	0.998	0.852
MELD score	16.4	0.596	0.875	0.730	0.184	0.988	0.731
CTP points	9.5	0.542	0.958	0.584	0.142	0.995	0.609

CLIF-SOFA, chronic liver failure-sequential organ failure assessment; MELD, model for end-stage liver disease; CTP, Child–Turcotte–Pugh; PPV, positive predictive value; NPV, negative predictive value

Since the history of hepatocellular carcinoma was one of important risk factors for 6-week mortality, we made further subgroup ROC analysis depends on with or without a history of hepatocellular carcinoma in Table 4.

**Table 4:** CLIF-SOFA score according to history of HCC.

**Area under the receiver operator characteristics (AUROC)**										
**6-week mortality**										
Variable		Mortality rate (%)			AUROC		95% CI			P-value
All (n = 714)		13.1			0.943		0.917–0.968			<0.001
HCC (n = 146)		21.2			0.964		0.925–1.0			<0.001
Non-HCC (n = 568)		10.9			0.937		0.905–0.970			<0.001
**Hospital mortality**										
Variable		Mortality rate			AUROC		95% CI			P-value
All		6.9			0.964		0.949–0.979			<0.001
HCC		8.9			0.956		0.923-.0990			<0.001
Non-HCC		6.2			0.965		0.947–0.983			<0.001
**Cut-off points according to Youden index**										
** 6-week mortality**										
Predictive factors	Cut-off point		Youden index	Sensitivity (%)		Specificity (%)		PPV	NPV	Accuracy
CLIF-SOFA	6.5		0.758	0.871		0.887		0.536	0.979	0.885
HCC	6.5		0.834	0.903		0.930		0.778	0.973	0.924
Non-HCC	5.5		0.742	0.968		0.775		0.345	0.995	0.795
** Hospital mortality**										
CLIF-SOFA	6.5		0.823	0.979		0.844		0.311	0.998	0.852
HCC	6.5		0.827	1.0		0.827		0.361	1.0	0.842
Non-HCC	6.5		0.819	0.971		0.848		0.296	0.998	0.856

CLIF-SOFA, chronic liver failure-sequential organ failure assessment; HCC, hepatocellular carcinoma

In order to predict 6-week mortality, the cut-off point was 6.5 for patients with hepatocellular carcinoma. The result was similar with all patients. However, the cut-off point was changed to 5.5 for patients without hepatocellular carcinoma.

In order to predicting hospital mortality, the cut-off point was 6.5 for patients with or without hepatocellular carcinoma. The result was the same with all patients.

## Discussion

Our study demonstrated that CLIF-SOFA score was a good predictive system for not only hospital but also 6-week mortality of post successful endoscopic variceal ligation. There are several predictive models for upper gastrointestinal bleeding such as Rockall score (RS), Glasgow Blatchford Score (GBS), Baylor Bleeding score, Cedars-Sinai Medical Center predictive index, Almela score, and AIMS65 score[13–17]. Thanapirom et al. showed significantly lower AUROC for prediction of mortality and re-bleeding in variceal bleeding compared with nonvariceal bleeding among full RS (0.57 vs. 0.80), pre-endoscopic RS (0.63 vs. 0.76), and GBS (0.63 vs. 0.66)[18]. Budimir et al. revealed AUROC for predicting variceal bleeding mortality among AIMS65, GBS and PRS were 0.74, 0.60, 0.67 respectively[19]. By contrast, Al-Freah et al. showed higher AUROC for predicting ICU hospital mortality variceal bleeding by CLIF-SOFA score (0.823), MELD score (0.839), and modified number of failed organs (0.843)[20]. In our cohort, CLIF-SOFA score predicted well for post EVL 6-week mortality as AUROC reached 0.943. According to the current literature, it seems that scoring systems consisting of liver function evaluation such as CLIF-SOFA and MELD scores predicted mortality more precisely than RS and GBS, which were used widely for general upper gastrointestinal bleeding.

Under pairwise comparison of area under the receiver operator characteristics, we indicated that the predicting power of CLIF-SOFA score was better than MELD score and CTP points for 6-week mortality of EVH post EVL in significant difference. The result could be explained by our multivariate hazard Cox analysis with parameters of scoring systems excluded. First, in addition to levels of serum bilirubin and creatinine, grade of hepatic encephalopathy, MAP, and respiration parameter SpO2/FiO2; these additional parameters were included in CLIF-SOFA score compared with MELD score. On the other hand, CLIF-SOFA score included five risk factors (grade of hepatic encephalopathy, SpO2/FiO2, MAP, serum bilirubin and creatinine) more than CTP points included two risk factors (serum albumin and grade of hepatic encephalopathy). We also considered that EVH was an acute on chronic event. Thus, CLIF-SOFA score included not only relatively chronic parameters of the liver like MELD score and CTP points but also relative acute parameters such as SpO2/FiO2 and MAP.

In addition, hepatocellular carcinoma was also an independent risk factor for 6-week mortality of EVH post EVL according to multivariate hazard Cox analysis with parameters of scoring systems included. This finding was the same as previous studies, and portal thrombosis induced advanced portal hypertension was suggested as the reason[21–23]. So, the cut-off point for predicting 6-week mortality was different after subgroup analysis according to with or without a history of hepatocellular carcinoma. However, hepatocellular carcinoma was not a risk factor for hospital mortality according to hazard Cox analysis in our study. Thus, the cut-off point for predicting hospital mortality was not different after subgroup analysis.

According to our cohort, we suggested that patients (whether with or without hepatocellular carcinoma) with CLIF-SOFA score ≥7 to be more closely monitored after EVL because of higher hospital mortality. Caring for them in the ICU is one of rationales, and besides, it may reduce medical expenses to care for relatively stable patient with CLIF-SOFA score below cut-off point in the ward. On the other hand, for hospital survivors with CLIF-SOFA score ≥6 (without history of hepatocellular carcinoma) or CLIF-SOFA score ≥7 (with history of hepatocellular carcinoma), earlier treatment goal discussion such as liver transplantation or hospice care were considered due to higher 6-week mortality.

There are some strengths in our study. First, a relatively large sample size was included because our hospital is the only referring center for EVL in Taitung (a remote southeastern county in Taiwan). Second, this was the first study to evaluate EVH purely managed by EVL according to CLIF-SOFA score. Third, our overall six-week mortality was 13.1% in agreement with the published literature[24]. It also confirmed recent improvement of prognosis of EVH[4, 20, 24, 25].

However, several limitations of the current study should be mentioned. First, our study was a retrospective observational study from a single hospital. Second, we did not compare CLIF-SOFA score with other scores such as acute physiology and chronic health evaluation (APACHE) or original sequential organ failure assessment (SOFA) score. Because not all patients with EVH post-EVL were cared for in the ICU, there was insufficient data for other scores frequently used in ICU. Third, the positive and negative predictive values were based on the mortality rate in our study. These values would be changed while applied in other hospital due to different mortality.

## Conclusions

CLIF-SOFA score predicts post-EVL prognosis well and may be an objective reference for physician’s decision-making. For patients without hepatocellular carcinoma, CLIF-SOFA score ≥6 suggests higher 6-week mortality and CLIF-SOFA score ≥7 suggests higher hospital mortality. For patients with hepatocellular carcinoma, CLIF-SOFA score ≥7 suggests higher 6-week and hospital mortality.

## Supporting information

S1 Table The chronic liver failure- sequential organ failure assessment (CLIF-SOFA) score. (DOCX)

S2 Table Cox analysis for 6-week mortality. (DOC)

S3 Table Cox analysis for hospital mortality. (DOC)

S4 Table Original individual data. (XLSX)
