# Supplementary material for: Application of chronic liver failure-sequential organ failure assessment score for the predication of mortality after esophageal variceal hemorrhage post endoscopic ligation
Source: PLoS One. 2017 Aug 2;12(8):e0182529. doi: 10.1371/journal.pone.0182529 (PMC5540601; doi:10.1371/journal.pone.0182529)
Supplement: S1 Table — (DOCX) [file pone.0182529.s001.docx]

| **S1 Table. The chronic liver failure- sequential organ failure assessment (CLIF-SOFA) score** | | | | | |
| --- | --- | --- | --- | --- | --- |
| **CLIF-SOFA score** | **0** | **1** | **2** | **3** | **4** |
| **Respiration** | | | | | |
| PaO2/FiO2  or SpO2/FiO2 | >400  >512 | >300 to ≤400  >357 to ≤512 | >200 to ≤300  >214 to ≤357 | >100 to ≤200  89 to ≤214 | ≤100  ≤89 |
| INR | <1.1 | ≥1.1 to <1.25 | ≥1.25 to <1.5 | ≥1.5 to <2.5 | ≥2.5 or  platelet ≤20 |
| **Liver** | | | | | |
| Bilirubin, mg/dL  (lmol/L) | <1.2 (<20) | ≥1.2 to <2.0  (20–32) | ≥2.0 to <6.0  (33–101) | ≥6.0 to <12.0  (102–204) | ≥12.0 (>204) |
| **Cardiovascular** | | | | | |
| Hypotension | MAP ≥70 mmHg | MAP <70 mmHg | Dopamine ≤5 or  dobutamine  (any dose)* or  terlipressin | Dopamine >5 or  epi ≤0.1 or  norepi ≤0.1* | Dopamine >15 or  epi >0.1 or  norepi >0.1* |
| **CNS** | | | | | |
| HE grade | No HE | I | II | III | IV |
| **Renal** | | | | | |
| Creatinine  (mg/dL) | <1.2 | ≥1.2 to <2.0 | ≥2.0 to <3.5 | ≥3.5 to <5.0 or  use of RRT | ≥5.0 |
| CNS, central nervous system; epi, epinephrine; FiO2, fractional inspired oxygen; HE, hepatic encephalopathy; INR, international normalized ratio; MAP, mean arterial pressure; norepi, norepinephrine, PaO2, arterial oxygen tension; RRT, renal replacement therapy; SpO2, pulse oximetric saturation | | | | | |
| * Adrenergic agents administered for at least 1 h (doses are given in lg/kg/min). | | | | | |
