# Supplementary material for: Application of chronic liver failure-sequential organ failure assessment score for the predication of mortality after esophageal variceal hemorrhage post endoscopic ligation
Source: PLoS One. 2017 Aug 2;12(8):e0182529. doi: 10.1371/journal.pone.0182529 (PMC5540601; doi:10.1371/journal.pone.0182529)
Supplement: S2 Table — (DOC) [file pone.0182529.s002.doc]

| **S2 Table. Cox analysis for 6-week mortality** | | | |
| --- | --- | --- | --- |
| **Parameter** | **Hazard ratios** | **95% CI** | **P-value** |
| **Univariate hazard analysis** | | | |
| History of HCC | 2.207 | 1.317-3.119 | 0.001 |
| MAP | 0.969 | 0.959-0.979 | <0.001 |
| Hemoglobin | 0.859 | 0.788-0.936 | 0.001 |
| Leucocytes | 1.000 | 1.000-1.000 | <0.001 |
| Bilirubin | 1.100 | 1.079-1.122 | <0.001 |
| Prothrombin time INR | 1.167 | 1.032-1.320 | 0.014 |
| Albumin | 0.385 | 0.275-0.541 | <0.001 |
| Creatinine | 1.289 | 1.220-1.363 | <0.001 |
| Platelets | 1.000 | 1.000-1.000 | 0.002 |
| Hepatic encephalopathy | 2.345 | 2.018-2.726 | <0.001 |
| SpO2/FiO2 | 0.991 | 0.989-0.994 | <0.001 |
| CPT points | 1.998 | 1.771-2.255 | <0.001 |
| MELD score | 1.102 | 1.086-1.119 | <0.001 |
| CLIF-SOFA score | 1.659 | 1.565-1.758 | <0.001 |
| **Multivariate hazard analysis** | | | |
| **Model excluding scoring systems** | | | |
| History of HCC | 2.212 | 1.407-3.478 | 0.001 |
| MAP | 0.983 | 0.969-0.998 | 0.024 |
| Bilirubin | 1.070 | 1.046-1.095 | <0.001 |
| Albumin | 0.500 | 0.331-0.757 | 0.001 |
| Creatinine | 1.171 | 1.098-1.249 | <0.001 |
| Hepatic encephalopathy | 1.964 | 1.661-2.322 | <0.001 |
| SpO2/FiO2 | 0.996 | 0.993-0.998 | 0.001 |
| **Model including scoring systems** | | | |
| CLIF-SOFA score | 1.702 | 1.335-2.170 | <0.001 |
| History of HCC | 2.507 | 1.590-3.954 | <0.001 |
| HCC, hepatocellular carcinoma; MAP, mean arterial pressure; INR, international normalized ratio; SpO2, pulse oximetric saturation; FiO2, fractional inspired oxygen; CTP, Child–Turcotte–Pugh; MELD, model for end-stage liver disease; CLIF-SOFA, chronic liver failure-sequential organ failure assessment. | | | |
