# Supplementary material for: Application of chronic liver failure-sequential organ failure assessment score for the predication of mortality after esophageal variceal hemorrhage post endoscopic ligation
Source: PLoS One. 2017 Aug 2;12(8):e0182529. doi: 10.1371/journal.pone.0182529 (PMC5540601; doi:10.1371/journal.pone.0182529)
Supplement: S3 Table — (DOC) [file pone.0182529.s003.doc]

| **S3 Table. Cox analysis for hospital mortality** | | | |
| --- | --- | --- | --- |
| **Parameter** | **Hazard ratios** | **95% CI** | **P-value** |
| **Univariate hazard analysis** | | | |
| History of HCC | 0.680 | 0.360-1.285 | 0.235 |
| MAP | 0.960 | 0.948-0.972 | <0.001 |
| Hemoglobin | 0.836 | 0.740-0.944 | 0.004 |
| Leucocytes | 1.000 | 1.000-1.000 | <0.001 |
| Bilirubin | 1.113 | 1.087-1.139 | <0.001 |
| Prothrombin time INR | 1.197 | 1.027-1.395 | 0.021 |
| Albumin | 0.375 | 0.233-0.602 | <0.001 |
| Creatinine | 1.294 | 1.218-1.376 | <0.001 |
| Hepatic encephalopathy | 2.269 | 1.847-2.787 | <0.001 |
| Ascites | 1.945 | 1.044-3.624 | 0.036 |
| SpO2/FiO2 | 0.991 | 0.988-0.994 | <0.001 |
| CPT points | 2.076 | 1.752-2.458 | <0.001 |
| MELD score | 1.116 | 1.095-1.138 | <0.001 |
| CLIF-SOFA score | 1.715 | 1.583-1.858 | <0.001 |
| **Multivariate hazard analysis** | | | |
| **Model excluding scoring systems** | | | |
| MAP | 0.973 | 0.954-0.992 | 0.007 |
| Bilirubin | 1.094 | 1.057-1.132 | <0.001 |
| Albumin | 0.487 | 0.251-0.942 | 0.033 |
| Creatinine | 1.134 | 1.050-1.224 | 0.001 |
| Hepatic encephalopathy | 1.839 | 1.440-2.349 | <0.001 |
| SpO2/FiO2 | 0.996 | 0.993-0.999 | 0.020 |
| **Model including scoring systems** | | | |
| CLIF-SOFA score | 1.742 | 1.220-2.486 | 0.002 |
| Prothrombin time INR | 0.332 | 0.120-0.923 | 0.035 |
| HCC, hepatocellular carcinoma; MAP, mean arterial pressure; INR, international normalized ratio; SpO2, pulse oximetric saturation; FiO2, fractional inspired oxygen; CTP, Child–Turcotte–Pugh; MELD, model for end-stage liver disease; CLIF-SOFA, chronic liver failure-sequential organ failure assessment. | | | |
